# Overdose prevention for prisoners in New York: a novel program and collaboration

**DOI:** 10.1186/s12954-015-0084-8

**Published:** 2015-11-05

**Authors:** Howard Zucker, Anthony J. Annucci, Sharon Stancliff, Holly Catania

**Affiliations:** New York State Department of Health, Corning Tower, Empire State Plaza, Albany, NY 12237 USA; New York State Department of Corrections and Community Supervision, The Harriman State Campus, Bldg. 2, 1220 Washington Avenue, Albany, NY 12226-2050 USA; Harm Reduction Coalition, 22 W 27th St Fl 5, New York, NY 10001 USA; New York State Department of Health AIDS Institute, 90 Church Street, 13th floor, New York, NY 10007 USA

**Keywords:** Opioid overdose prevention, Prisoner re-entry, Pre-release prisoner training, Prisoner overdose fatality, Naloxone, Overdose prevention with naloxone, Public health, Prisoner health, Opioid deaths

## Abstract

This is a brief report on the establishment of a new program in New York State prisons to prepare prisoners to avoid the increased risks of drug overdose death associated with the transition to the community by training them in overdose prevention and making available naloxone, a medication that quickly reverses the effects of an opioid overdose, to all prisoners as they re-enter the community. It is a milestone collaboration in the USA between public health, the correctional system, and a community-based harm reduction program in response to the growth of heroin and opioid analgesic use and related morbidity and mortality, working together to get naloxone into the hands of the people at high risk of overdosing and/or of witnessing an opioid overdose.

## Background

Death from drug poisoning is a national issue in the USA. According to the Centers for Disease Control and Prevention, it is the leading cause of injury-related mortality in the USA and is associated with more than 40,000 lives lost annually. Over 50 % of drug poisoning deaths are attributed to opioids such as heroin and prescription of opioid analgesics. Heroin-related overdose deaths have nearly tripled across the country from 2010 to 2013 [1].

Opioid users who transition from confinement in prison or other institutional settings to the community are at particularly high risk for an overdose. Studies indicate that newly released prisoners have very high rates of drug overdose deaths [2].

## Main text

New York State’s response to the increase in heroin and opioid analgesic use, misuse, and deaths includes trainings in overdose recognition, and response and access to naloxone to reverse opioid overdoses, a practice first implemented in 2006. As of this writing, there are more than 225 registered overdose prevention programs across the state that have trained more than 75,000 overdose responders—including people who use drugs, their families and friends, law enforcement officers, treatment providers and others who may be likely to witness overdoses or be first on the scene to respond to them—and reversed over 1800 overdoses [3]. The Department of Health anticipates the number of registered programs will grow to more than 240 by the end of 2015.

Building on a community overdose prevention model, the department has formed and strengthened collaborations with community partners, and state and local government agencies whose work focuses on areas such as health, behavioral health, public safety, and education.

The goal is to ensure that those who either witness an overdose or who are first on the scene to respond have appropriate training to keep victims alive until they can receive medical attention.

In February 2015, the New York State Department of Health AIDS Institute (DOHAI), New York State Department of Corrections and Community Supervision (DOCCS), and the Harm Reduction Coalition (HRC) launched a novel opioid overdose prevention and training program, preparing inmates for reentry into the community. This program will target soon to be released inmates in all 54 state correctional facilities and educate them about the risks of opioid use, especially after periods of confinement, and train them in the use of naloxone. Naloxone, intended for intranasal use, will be offered to trained inmates free of charge at release [4].

A pilot at a minimum-security correctional facility in New York City was initiated in February 2015. Harm Reduction Coalition staff trained inmates in the use of naloxone, as well as prison staff who can now provide the trainings. As of September 2015, more than 700 inmates have been trained at Queensboro Correctional Facility; about 200 have received kits. The numbers of inmates taking kits at release has increased each month, suggesting growing acceptance of the program. Training has been initiated in two other correctional facilities and several others have scheduled staff trainings. In addition, a community-based organization in the region is training family members and friends of incarcerated individuals and equipping them with naloxone free of charge.

DOCCS has also established a statewide standing order, in conjunction with the Department of Health, which enables DOCCS nursing staff to administer naloxone by injection to any inmate, staff or visitor suspected of an overdose without first obtaining a physician order. Previously, DOCCS staff had to seek individual doctor orders when an overdose was suspected.

## Conclusion

To complement these efforts, parole officers are now also being trained. Since parolees and others under criminal justice supervision are not permitted to associate with people who use drugs, nor use drugs themselves, it is important that parole departments and others in the criminal justice system buy into the program and prioritize saving lives. Acceptance of the program has been augmented by the fact that many corrections staff and parole officers recognize the need for naloxone in their communities. As a result, they have been eager to take their knowledge and naloxone home with them.

## Abbreviations

DOCCS: Department of Corrections and Community Supervision of the State of New York
DOHAI: New York State Department of Health AIDS Institute
HRC: Harm Reduction Coalition
